# Changes in markers for cardio-metabolic disease risk after only 1-2 weeks of a high saturated fat diet in overweight adults

**DOI:** 10.1371/journal.pone.0198372

**Published:** 2018-06-27

**Authors:** Jeffrey F. Horowitz, Juan F. Ortega, Alexander Hinko, Minghua Li, Rachael K. Nelson, Ricardo Mora-Rodriguez

**Affiliations:** 1 School of Kinesiology, University of Michigan, Ann Arbor, Michigan, United States of America; 2 Exercise Physiology Lab, University of Castilla-La Mancha, Toledo, Spain; 3 College Health Professions, Health Sciences Department, Central Michigan University, Mt. Pleasant, Michigan, United States of America; Universidad Europea de Madrid, SPAIN

## Abstract

**Purpose:**

Diets high in saturated fat acids (SFA) have been linked with cardio-metabolic disease risk. The purpose of this study was to determine whether only 1–2 weeks of a high SFA diet could impact disease risk factors in overweight adults who normally eat a relatively low proportion of SFA (i.e., <40% of dietary fat).

**Methods:**

Twelve overweight (BMI: 27±1 kg/m^2^) young adults were studied before and after a 2-week diet that increased the proportion of SFA (<40% to 60% of dietary fat), while maintaining their daily intake of total fat, carbohydrate, protein, and calories. Insulin resistance, blood pressure, plasma markers of liver damage, total plasma cholesterol concentrations, and fatty acid profile within plasma and skeletal muscle lipid pools were assessed before and after the intervention.

**Results:**

Total plasma cholesterol concentration increased (148±5 *vs*. 164±8 mg/dl; P<0.05) after only one week, due exclusively to an increase in LDL-cholesterol (78±4 *vs*. 95±7 mg/dl; P<0.05). After two weeks, plasma aspartate amino transferase (AST) concentration increased (P<0.05) but we found no change in insulin resistance, or resting blood pressure. The diet increase the proportion of SFA in plasma (35±1% *vs*. 39±2%; P<0.05) and the intramyocellular triglyceride pool (32±1% *vs*. 37±1%; P<0.05) suggesting the fatty acids in these pools may readily exchange.

**Conclusions:**

Although blood lipids remain within normal clinical range, increasing saturated fat in diet for only 2 weeks raises plasma markers of cardiovascular risk (LDL-cholesterol) and liver damage (AST). In overweight, but healthy-young adults SFA accumulate in plasma and muscle after only 1–2 weeks of dietary increase.

## Introduction

Dietary prescriptions aimed at reducing cardio-metabolic disease risk often recommend modifying the amount and “type” of dietary fat (i.e., saturated, monounsaturated, and polyunsaturated fat). Although the impact of the type of dietary fat on disease risk is debated [1], there is considerable evidence linking diets high in saturated fat with an atherogenic blood lipid profile and insulin resistance [2–7]. Importantly, the time-course for measurable changes in clinically relevant health outcomes in response to modifying the amount of dietary saturated and/or unsaturated fat remains unclear. Previously, we found that increasing the proportion of dietary saturated fats in subjects who typically ingest a diet relatively low in saturated fat, elevated plasma low-density lipoprotein cholesterol (LDL-C) concentration in just two weeks [8]. Although these subjects did not become hyperlipidemic, and their LDL-C did not reach the level linked to cardiovascular disease risk after the two-week diet, the fact that the increases in plasma LDL-C were statistically significant in such a short time is alarming.

The primary aim of the present study is to determine if this negative effect of a high saturated fat diet on blood lipid profile may be evident even earlier than 2 weeks, and to investigate if whole-body insulin action may also be affected by a very short exposure to a high saturated fat diet. Diets high in saturated fat may generate some negative health effects by increasing the incorporation of saturated fatty acids into various lipid pools. For example, increased saturated fatty acid incorporation in lipid pools within insulin responsive tissues (e.g., skeletal muscle) may influence insulin resistance [9]. However, the time course for changes in fatty acid profile within skeletal muscle lipids is not well described. Moreover, it is not known if changes in the plasma fatty acid profile readily reflect in a change in muscle lipids pools. Therefore, a secondary aim of this study was to characterize changes in the fatty acid profile of different lipid species within plasma and skeletal muscle in response to short-term exposure (two weeks) of a high saturated fat diet.

## Materials and methods

### Subjects

Twelve overweight, but otherwise healthy (age: 24±2 years; body mass index 27.2±0.8 kg/m^2^) men (n = 10) and women (n = 2) volunteered for this study. Participants were not taking any medications known to affect their metabolism. All participants were non-smokers, women were not pregnant or lactating, and all subjects were weight stable (i.e., ± 2 kg) for at least 6 months prior to the study. Participants were not involved in any type of exercise program. Written informed consent was obtained from all participants prior to participation. All procedures were approved by the University of Michigan Institutional Review Board and the Ethics Committee at the Hospital Virgen de la Salud in Toledo (Spain).

### Study design

All participants completed an experimental trial before and again after a 2-week high saturated fat dietary intervention. The dietary intervention required subjects to exchange foods from their normal/habitual diet that were naturally high in unsaturated fat with foods high in saturated fats. The overall objective of the dietary intervention was to increase the proportion of saturated fat in their daily diet from <40% to 60% of their total fat intake, without changing total daily fat or energy intake. Before and after the 2-week diet period subjects arrived to the laboratory after an overnight fast. Nude body weight (Toledo, Metler, USA) was collected and 7-site skinfolds (Holtain, Tanner/Whitehouse, USA) measured to calculate body composition changes. Then, an intravenous catheter (BD Insyte, Becton Dickinson, Spain) was percutaneously inserted in an antecubital vein, and a 10 cc blood sample was collected for analysis of plasma concentrations of glucose, insulin, blood lipids, and liver enzymes (see details in “Plasma analytes” section, below). An OGTT started with the ingestion of 75 g of anhydrous glucose (Guinama Laboratorio, Spain) diluted into 250 mL of water. Immediately after all fluid was ingested, a timer was started and 5 mL blood samples were obtained every 15 min up to 120 min after glucose ingestion. The next day, biopsies samples were taken with suction from the *vastus lateralis* muscle under local anesthesia (2% lidocaine without epinephrine; Braun, Germany) using a modified Bergstrom needle. Muscle samples were rapidly cleaned of blood, fat and connective tissue, liquid N_2_ frozen and stored at -80°C until analysis. In addition to the OGTT and muscle biopsies collected before and after the 2-week dietary intervention, we also collected a fasting blood sample after just 1 week of the diet to assess whether the diet induced even more rapid changes in blood lipid profile. For both experimental trials, subjects abstained from any strenuous physical activity for at least 72 hours before the trials, otherwise they were asked to maintain their normal daily activities throughout the intervention.

### Diets

Participants completed a thorough food frequency questionnaire [10] to assess the content and composition of their habitual diets (Table 1). Subjects also completed a detailed food diary for the three days leading up to the first experimental trial, and they maintained their detailed daily food journals and a physical activity journal (IPAQ [11]) throughout the 2-week dietary intervention. Computerized dietary analysis of subject’s records (CESNID, Barcelona, Spain) was used to calculate energy intake, macronutrient composition, and dietary fat composition (i.e., saturated, monounsaturated, and polyunsaturated fat). All subjects were Spanish citizens and their habitual dietary patterns before the intervention resembled those previously reported for Spanish adults [12] which included a relatively high proportion of unsaturated fat (Table 1). During the 2-week intervention, we provided subjects with 1.2 g of dietary fat/kg body weight each day, while they abstained from their habitual dietary fat sources. The foods provided were largely whole-fat dairy products that contained a very high proportion of saturated fat. The amount of dietary fat provided was calculated to match their habitual dietary fat intake. Subjects returned to the laboratory every 1–3 days to be weighed, inspect their dietary and physical activity journals, and to pick-up their diet rations.

**Table 1:** Subjects’ daily habitual diet and the “study diet” (high saturated fat diet).

	Habitual diet	Study diet
**Daily energy intake** (kcals)	2415±226	2379±114.
**Fat** (g)	100±6.	99±5.
***Saturated fat*** *(%total fat)*	37±2%	60±1%*
***Unsaturated fat*** *(%total fat)*	63±2%	40±1%*
*Monounsaturated fat (%total fat)*	46±1%	32±1%*
*Polyunsaturated fat (%total fat)*.	17±1%	8±1%*
**Carbohydrate** (g)	248±23	232±16.
**Protein** (g)	119±12	120±9.

Values are means±SE.

* Significantly different from Habitual diet, P<0.05

### Analytical procedures

#### Plasma analytes

Plasma concentrations of glucose (glucose oxidase assay; Fisher Scientific), fatty acid (NEFA-HR assay kit; WAKO Chemicals USA), triglyceride (triglyceride reagent; Sigma Aldrich), total-cholesterol (Total-C) and high-density lipoprotein (HDL-C) (cholesterol oxidase reaction; BioSystems, Spain) were measured with commercially available colorimetric assay kits. Plasma insulin concentration was measured using a commercially available radioimmunoassay (RIA) kit (Human insulin RIA kit; Millipore). Plasma aspartate and alanine aminotransferase (AST and ALT) and C reactive protein (CRP) where analyzed using specific enzymatic kits (BioSystems, Spain) and a multichannel spectrometer plate reader (Versamax, Molecular Devices, USA).

#### Fatty acid species in muscle triglyceride, phospholipid, and plasma non-esterified fatty acid (NEFA)

Lipids were extracted from both plasma, and homogenized muscle samples using a single-phase mixture chloroform:methanol:saline (1:2:0.8) as previously described [13]. Internal lipid markers for triacylglycerol, diacylglycerol, monoacylglycerol, NEFA, phospholipid and cholesterol ester with fatty acid moieties of odd carbon number were added at the start of extraction, for subsequent purity and recovery determinations (NuChek; Avanti Polar Lipids). After chloroform separation, individual lipid species were eluted using specific solvent mixtures [14]. Fatty acid methyl esters (FAMES) were generated from purified glycerolipids in these samples while NEFA were converted to methyl esters [15]. FAMES were measured by gas chromatography and electron-impact mass spectrometry (Agilent 6890A GC and 5973N MSD), and quantified using FAME standards (NuChek).

### Calculations

#### Insulin sensitivity index (ISI)

We estimated insulin sensitivity during the OGTT using the Matsuda Composite Index [16] (also referred to as the “Insulin sensitivity Index” (ISI)).

$$ISI\;=\;10,000+\;\sqrt{\left(FPG\;x\;FPI)\;x\;(OGTT\;[glucose]\;x\;OGTT\;[insulin]\right)}$$

where FPG and FPI are fasting plasma glucose and insulin, respectively, and OGTT [glucose] and OGTT [insulin] represent the mean plasma glucose and insulin concentrations during the 2h OGTT procedure.

#### Homeostatic model assessment of insulin resistance (HOMA-IR)

Fasting plasma glucose and insulin were used to calculate HOMA-IR:

$$HOMA-IR\;=\;\frac{\left(FPG\;x\;FPI\right)}{22.5}$$

#### Low-density lipoprotein concentration

Low density lipoprotein cholesterol concentration (LDL-C) was calculated using the Friedewald equation [17].

$$LDL-C\;=\;Total-C-(HDL-C+\left(\frac{triacylglycerol}{5}\right))$$

#### Percent body fat

We calculated body density from the sum of skin folds using classic regression equations generated by Jackson and Pollock [18] and Durnin and Womersly [19] for both men and women. We used the mean body density calculated using both sets of equations and then percentage of body fat was calculated using the Siri equation.

$$\%Body\;fat\;=\;\left[\frac{495}{body\;density}-450\right]/100$$

### Statistical analysis

We used a one-way ANOVA with repeated measures and Tukey post hoc analysis when appropriate, for Total-C, LDL-C, and HDL-C, which were measured at three different time points during the study (i.e., before, at 1 week and after 2 weeks of the intervention). All other outcomes were only measured before and after the intervention, and we used the student’s paired t-test to assess statistical significance, which was set at *P* < 0.05. All data are presented as means ± SE.

## Results

### Study diet

Energy intake, total fat, carbohydrate, and protein intake of the high saturated fat diet were all well matched with the subjects’ habitual diet (Table 1). Although the amount of daily fat ingestion during the study was identical to the subjects’ habitual diet, the subjects ate nearly 60% more saturated fat (and nearly 40% less unsaturated fat) during the study (Table 1).

### Body composition and physical activity

Body weight decreased very slightly (<1%), but because all subjects either maintained weight or experience a small reduction in body weight, this reductions in body mass was statistically significant after the two-week dietary intervention (Table 2). In contrast, percent body fat, fat mass, and fat free mass did not change significantly with the diet intervention (Table 2). Subjects’ habitual physical activity did not change during the 2 week study according to analysis of their activity journals (IPAQ; Table 2). In general, their IPAQ values (MET-min·week^-1^) were similar to those previously reported for Spanish adults in the same age range [20].

**Table 2:** Subject characteristics before and after the 2-week high saturated fat diet.

	BEFORE	AFTER
**Body weight** (kg)	82.3±2.9	81.5±2.9*
**Body mass index** (kg/m^2^)	27.2±0.8	27.0±0.8*
**Percent body fat** (%)	18.5±2.1	18.2±2.0
**Fat mass** (kg)	15.2±1.7	14.8±1.8
**Fat free mass** (kg)	67.1±3.0	66.8±3.0
**Physical activity IPAQ** (MET-min·week^-1^)	2141±211	2201±264

Values are means±SE.

* Significantly different from BEFORE, P<0.05

### Clinical outcome measures

**Fig 1:**
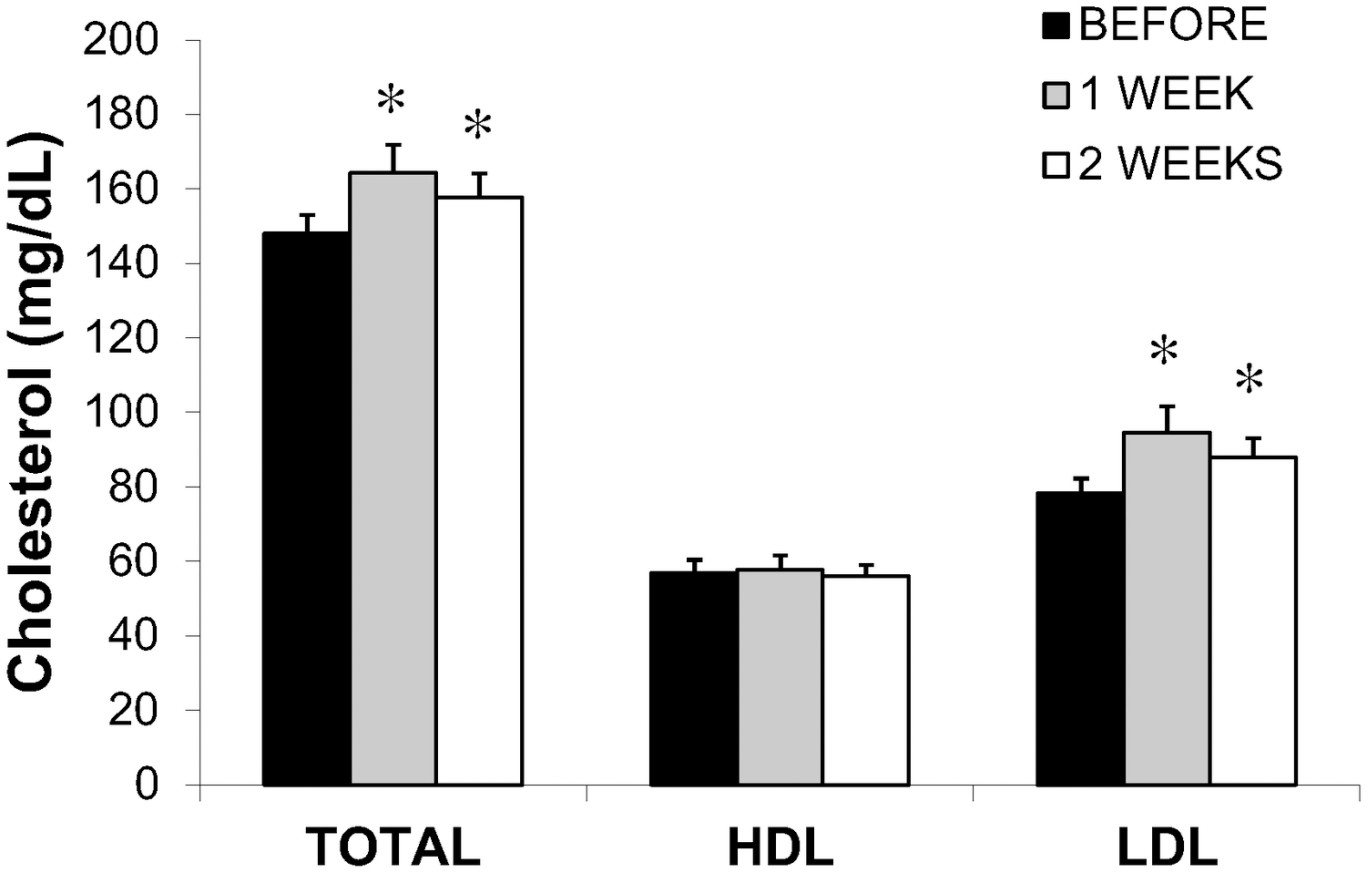
Plasma cholesterol concentrations before the dietary intervention and again after 1 week and 2 weeks of the high saturated fat diet intervention. Values are means±SE. * Significant difference from BEFORE, P<0.05. Total cholesterol (Total-C), high density lipoprotein colesterol (HDL-C), and low density lipoprotein cholesterol (LDL-C).

Plasma total cholesterol concentration was significantly elevated after only one week of the Study diet (P<0.05), and remained elevated at week 2 (P<0.05) (Fig 1). This increase in total cholesterol was due to an elevation in plasma LDL-C concentration, while HDL-C remained unchanged during the two weeks of the dietary intervention (Fig 1).

**Fig 2:**
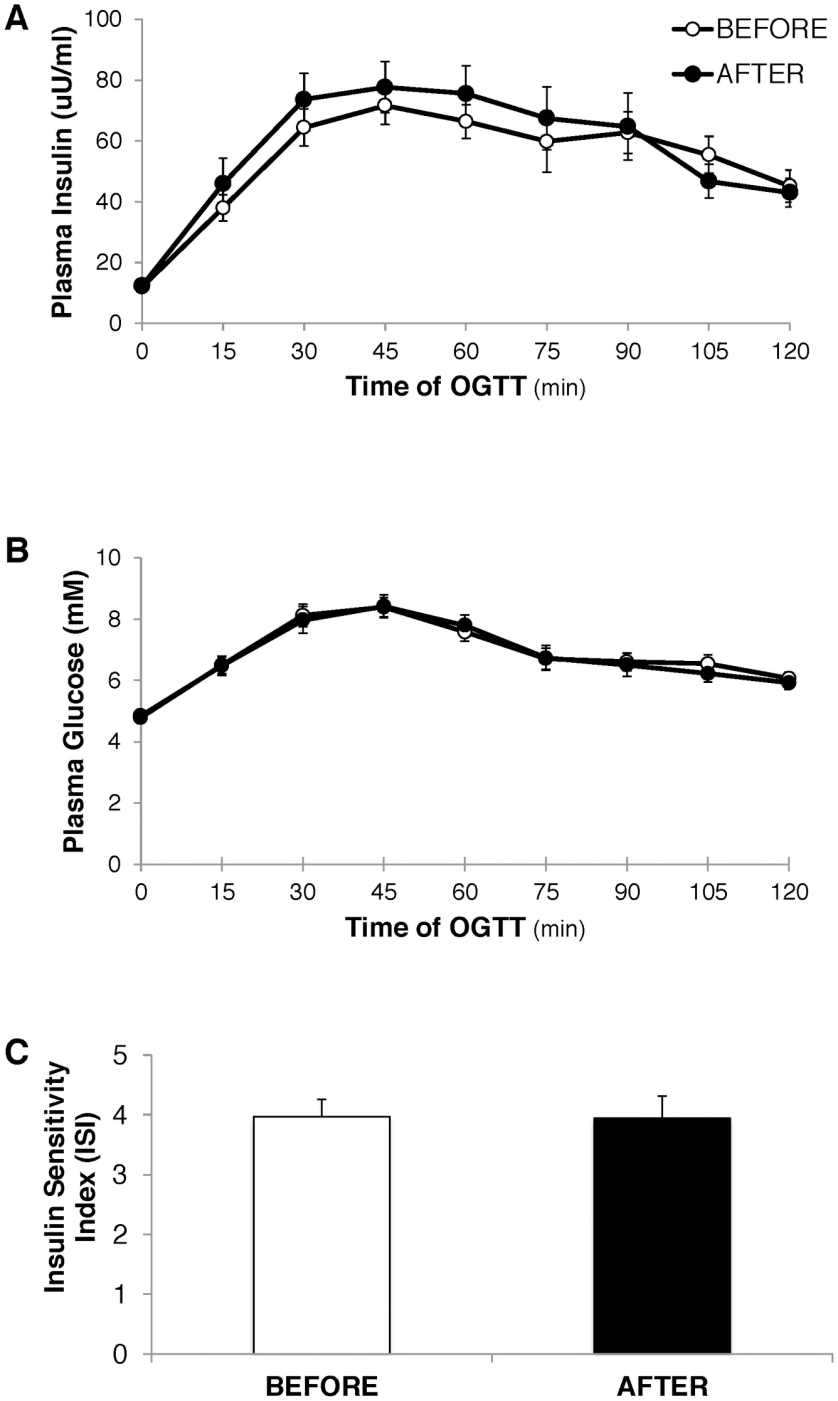
Responses during the oral glucose tolerance (OGTT) before at after the 2 week high saturated fat diet intervention. A. Plasma insulin concentration, B. Plasma glucose concentration, and C. “Insulin sensitivity index” (Matsuda Composite Index) calculated from plasma insulin and glucose concentrations during the OGTT.

The relatively short-term exposure to the high saturated fat diet also increase plasma concentration of the liver enzyme, AST (Table 3), which provides a crude marker for hepatic steatosis. In contrast, the insulin and glucose responses during the OGTT were nearly identical before *vs*. after the diet (Fig 2A and 2B), and as a result, our assessment of insulin sensitivity (Insulin Sensitivity Index (ISI)) was also not affected by a 2-week exposure to the high saturated fat diet (Fig 2C).

**Table 3:** Fasting plasma substrate and hormone concentrations and other clinical measures before and after the high saturated fat diet intervention.

	BEFORE	AFTER
**Glucose concentration** (mM)	4.8±0.1	4.8±0.1
**Insulin concentration** (μIU/ml)	13±1	12±1
**HOMA-IR**	2.62±0.19	2.75±0.34
**Fatty acid concentration** (mM)	0.43±0.07	0.45±0.07
**Triacylglycerol concentration** (mM)	0.74±0.11	0.79±0.09
**C-reactive protein** (μg/ml)	0.5±0.1	0.6±0.2
**AST** (U/L)	28.2±2.8	35.4±3.5*
**ALT** (U/L)	29.7±2.8	32.7±3.7
**Systolic BP** (mmHg)	119±3	125±4.
**Diastolic BP** (mmHg)	72±2	75±2

Values are means±SE. AST: aspartate transaminase; ALT: alanine transaminase; HOMA-IR: Homeostatic Model Assessment of Insulin Resistance.

* Significantly different from BEFORE, P<0.05.

Similarly, resting blood pressure, as well as fasting plasma concentrations of glucose, insulin, fatty acid, triacylglycerol, and C-reactive protein (a crude index of systemic inflammation) were also not affected by the 2-week high saturated fat diet (Table 3).

### Plasma and muscle fatty acid profiles

The two weeks high saturated fatty acid diet did not affect concentrations of triacylglycerol or phospholipids in skeletal muscle (i.e., intramuscular phospholipids; IMPL), and both remained remarkably stable throughout the intervention (IMTG: 10.2±1.8 *vs*. 10.2±2.8 and IMPL: 6.3±0.2 *vs*. 6.3±0.1 nmol/mg wet weight before *vs*. after the 2 week high saturated fat diet, respectively). However, the high saturated fat diet did significantly increase the proportion of saturated fatty acids in the plasma fatty acid pool, with a similar increase in the proportion of saturated fatty acids in the IMTG pool (Table 4). Conversely, the proportion of unsaturated fatty acids decreased in these fractions, almost exclusively due to a reduction in the proportion of polyunsaturated fatty acids (Table 4). Interestingly, the percentage of saturated fatty acids within IMPL pool did not increase with the high saturated fat diet. In fact, we observed a very slight, yet significant reduction (P<0.05) in the proportion of saturated fatty acids in IMPL, along with a compensatory increase (P<0.05) in the proportion of polyunsaturated fatty acids in IMPL after the high saturated fat diet intervention (Table 4). Details of the specific fatty acid profiles within these fractions is available in supplemental table 1 (S1 Table).

**Table 4:** Fatty acid composition in diet, plasma pool, muscle triacylglycerol and phospholipid fractions before and after 2 weeks of a diet high in saturated fat.

		Dietary fat	Plasma Fatty acid	Muscle triacylglycerol	Muscle phospholipid
**Saturated Fatty acids**	BEFORE	37±2%	35±1%	32±1%	44±1%
	AFTER	66±1%*	39±2%*	37±1%*	43±1%*
**Unsaturated Fatty acids**	BEFORE	63±2%	65±1%	68±1%	56±1%
	AFTER	34±1%*	61±2%*	63±1%*	57±1%*
**Polyunsaturated**	BEFORE	17±1%	18±1%	18±1%	46±1%
	AFTER	9±1%*	15±1%*	15±1%*	48±1%*
**Monounsaturated**	BEFORE	46±1%	47±1%	50±1%	10±1%
	AFTER	25±1%*	46±1%	48±1%	9±1%*

Values are means±SE.

* Significantly different from BEFORE, P<0.05.

## Discussion

A key clinically relevant finding from the present study was that plasma total cholesterol and LDL-C increased after only 1 week of the isocaloric high saturated fat diet. Additionally, we found that a clinical marker of liver damage was measurably elevated after our 2-week high saturated fat diet intervention. Together these findings suggest that even a very short-term exposure of a high saturated fat diet can significantly increase markers of disease risk factors in overweight young adults. It is important to note that these rapid changes in disease risk in response to the high saturated fat diet were observed despite a very slight but significant weight loss. Interestingly, we also found that the increased proportion of saturated fatty acids within the plasma fatty acid pool was nearly identical to the increase found in the intramyocellular triacylglycerol pool. In contrast, the fatty acid profile within the intramyocellular phospholipid pool was “protected” from an increase in saturated fatty acids. Our data reveals that two weeks increase in dietary saturated fat raises plasma proportions of saturated fat and triacylglycerol composition in skeletal muscle which could potentially affect insulin actions in this tissue.

It has been well-described that diets high in saturated fatty acids augment plasma total cholesterol and LDL-C cholesterol [2, 4, 6, 7, 21]. We have reported that under similar conditions, plasma LDL-C increased after just 2 weeks of a high saturated fat diet [8] and others find similar results after 3 weeks [22]. The present finding that LDL-C increased after only one week of the high saturated fat diet is even more alarming. It appears that the increase in LDL synthesis may not be responsible for the increased LDL-C concentration found after exposure to high dietary saturated fatty acids [23, 24]. Evidence suggests that LDL receptor activity can be suppressed by diets high in saturated fat [25], reducing LDL-C clearance and thereby increasing LDL-C accumulation in the circulation. Impaired LDL receptor function may be due to reduced cell membrane fluidity stemming from high saturated fatty acid availability [25, 26]. Therefore, daily exposure to a high abundance of saturated fatty acids may underlie acute modifications in receptor function, which may help explain the rapid increase in LDL-C found with our dietary intervention.

Our finding that plasma AST concentration increased after 2 weeks of the high saturated fat diet, provides a crude indication of elevated hepatic stress, perhaps in consequence to increased accumulation of liver fat. Because of limitations in the ability to directly test liver fat content and liver function in human subjects, controlled experiments in animal models may be best suited to assess the impact of saturated fat on liver dysfunction. It has been found that increasing dietary saturated fat in rats (without increasing total energy intake) augmented hepatic triacylglycerol content after only one week, while plasma AST concentration was elevated by the fourth week of the diet. The authors also reported a concomitant increase in saturated fats into the endoplasmic reticulum (ER) membrane, which results in ER stress [27, 28]. Although our intervention did not yield profound alterations in either blood lipid profile or marker of liver damage, the fact the we did find measurable increases in plasma LDL-C and AST in only 1–2 week exposure to a high saturated fat diet without increasing total fat content suggests that the type of dietary fat may be an important contributor to long-term hepatic health.

Much epidemiological evidence suggests that diets high in saturated fatty acids are associated with a greater degree of insulin resistance [2, 29] but this finding is not universal [30, 31]. We acknowledge our finding that the high saturated fat diet did not impair our measurement of insulin sensitivity could certainly be a consequence of the dietary intervention being short, like in other investigations [32]. In addition, the use of OGTT to assess insulin sensitivity may not provide the resolution required to detect impairments in insulin action [33]. However, available evidence from intervention studies that provided a longer dietary intervention period and used more accurate measures of assessing insulin action are still equivocal [9, 34, 35]. Overall, it appears that many well-controlled intervention studies do not all support the notion that diets high in saturated fats induce insulin resistance [30]. Importantly, Vessby, et al., [9] reported impaired insulin sensitivity after 3 months of a diet high in saturated fat, but this effect was not observed in their participant with a total dietary fat content greater than 37% of total daily energy intake. Similarly, Jebb et al., [36] reported that in subjects ingesting more than 38% of their daily energy intake from fat, replacing dietary saturated fats with unsaturated fats for 6 months did not improve insulin sensitivity. In our subjects, fat represented 39% of their energy intake, which may help to explain why dietary substitution of unsaturated by saturated fats did not impair our assessment of insulin action. Although daily dietary fat intake approaching 40% of total energy is relatively high, this is often found to be close to the norm in many countries, including the United States [37, 38] and Spain [39]. Therefore, with a relatively high, yet normal daily fat intake, consuming a relatively high proportion of saturated fat during 2 weeks does not appear to have much/any impact on insulin sensitivity.

Our high saturated fat diet increased the proportion saturated fatty acids in plasma. However, the ~10% increase in the proportion of saturated plasma fatty acids was very small when compared with the nearly 80% increase in dietary saturated fat we implemented with the diet. Importantly, because we measured fatty acid profile in the overnight fasted condition, the fatty acids measured in the circulation were largely derived from endogenous triacylglycerol stored in adipose tissue. As such, the disparity between the fatty acid profiles within the diet and the plasma fatty acid pool is likely a consequence of dilution of the dietary fat within the fatty acids stored in adipose tissue, which are very large and very slow to turnover. In fact, it has been estimated that the lipid within adipocyte turn over approximately every 1.6 years [40].

Despite the very slow absolute rate of turnover of triacylglycerol stored in adipose tissue, other endogenous stores of triacylglycerol can turnover at a relatively high rate. Our finding that the proportion of saturated fatty acids within the plasma fatty acid pool were remarkably similar to that found in the intramyocellular triacylglycerol pool suggests that these lipid pools may exchange fatty acids quite readily [41]. Indeed, it was been suggested that all (or nearly all) fatty acids entering the muscle cell are initially stored as triacylglycerol, and then differentially metabolized within the cell [42, 43]. Our finding supports the idea that these two lipid pools are closely coupled. We did not measure muscle diacylglycerol concentration in this study, but it has been reported that similar to IMTG, the abundance of saturated fatty acids in diacylglycerides also increase in response to diets high in saturated fat [44]. In contrast to our findings in the IMTG pool, the fatty acid profile within the IMPL pool appeared to be largely protected from the dietary increases in saturated fat. This may reflect a relatively slow turnover of fatty acids within the muscle phospholipid pool or alternatively, the fatty acids within plasma and IMTG may not exchange readily with the IMPL pool. Phosphosolipids are the most abundant lipids found in cell membranes, and a relatively high proportion of saturated fatty acids within the phospholipid pool can reduce membrane fluidity, which in turn can impair cellular signaling [45–47]. Along these lines, elevated abundance of saturated fatty acids within skeletal muscle phospholipids have been linked with insulin resistance [48–50]. Therefore, our finding that 2 weeks of a high saturated fat diet did not rapidly increase the saturated fatty acid abundance in the IMPL pool may have contributed to our observation of unchanged insulin action after the diet intervention.

There were a number of limitations in our study that may confound our interpretation. Although we attempted to tightly control energy intake and energy expenditure subjects did lose on average 0.8 kg although no subject lost more than 2% of their initial body weight. Thus, we cannot rule out the possibility that this small weight loss may have counteracted some effects of the high saturated fat diet. Another limitation of our study was our assessment of insulin sensitivity. Although the Matsuda composite index matches tightly with clamp assessments [16], it is certainly possible that this method may not have been sensitive enough to detect relative small impairments in insulin action. Also, other than being overweight, our research participants were physically active and generally in good health. Our selective recruitment of this healthy subject population was a strategic decision for the study design, because this generally healthy group of subjects may be more susceptible to the negative effects of a high saturated fat diet. However, it is possible the degree of adiposity and physical fitness level of our participants may have masked some of the potential negative impact of the high saturated fat diet. In addition, the high saturated fat diet used in our study was largely derived from dairy products, which has been suggested may be more protective against insulin resistance compared with other sources of fat [51], however, this finding is not universal [52]. Finally, although we did not observe differences in any outcome between our male and female subjects, and the interpretation of our findings do not change if we remove the female subjects from our analyses, we still cannot rule out sex differences in the responses to a high saturated fat diet.

Overall, our findings highlight that diets high in saturated fat can induce rapid disarrangements in blood lipid profile and markers of hepatic health in as little as 1–2 weeks. Our finding that the high saturated fat diet changed fatty acid profile nearly identically within the plasma fatty acid pool and the intramyocellular lipid pool suggests that these lipid pools may readily and rapidly exchange fatty acids. In contrast, because the abundance of saturated fatty acids did not increase within the intramyocellular phospholipid pool, it appears that the muscle phospholipids may be somewhat protected from an increase in saturated fatty acids, which may in turn be helpful for the maintenance of membrane fluidity delaying the appearance of insulin resistance. In conclusion, although some important clinical markers (i.e., insulin sensitivity, blood pressure, fasting plasma glucose, and triacylglycerol concentrations) were not affected by a short-term increase in dietary saturated fat, our data reveals rapid increases in blood lipids (i.e., total cholesterol and LDL-C), liver damage markers, and the proportion of saturated fatty acids within plasma and muscle lipids, which over the long term may contribute to the development of metabolic and/or cardiovascular disease.

## Supporting information

S1 Table Proportion of specific fatty acid species measured in plasma, muscle triglycerides, and muscle phospholipid fractions. (PDF)
