# Supplementary material for: Changes in markers for cardio-metabolic disease risk after only 1-2 weeks of a high saturated fat diet in overweight adults
Source: PLoS One. 2018 Jun 27;13(6):e0198372. doi: 10.1371/journal.pone.0198372 (PMC6021040; doi:10.1371/journal.pone.0198372)
Supplement: S1 Table — (PDF) [file pone.0198372.s001.pdf]

Supplemental Table 1 Proportion of specific fatty acid species measured in plasma, muscle triglycerides, and muscle phospholipid fractions

	**Plasma fatty acids**		**Muscle triglycerides**		**Muscle Phospholipids**	
	**BEFORE**	**AFTER**	**BEFORE**	**AFTER**	**BEFORE**	**AFTER**
**C14:0**	2±1%	3±1%*	2±1%	4±1%*	<1%	<1%
**C16:0**	24±1%	27±1%*	24±1%	27±1%*	30±1%	29±1%*
**C18:0**	8±1%	8±1%	6±1%	6±1%	14±1%	13±1%*
**Total saturated**	35±1%	39±2%*	32±1%	37±1%*	44±1%	43±1%*
**C16:1**	3±1%	3±1%	3±1%	3±1%	<1%	<1%
**C18:1**	42±1%	40±1%	48±1%	45±1%*	9±1%	9±1%
**Total monounsaturated**	47±1%	46±1%	50±1%	48±1%	10±1%	9±1%*
**C18:2**	15±1%	12±1%*	17±1%	14±1%*	36±1%	36±1%
**C20:4**	2±1%	2±1%	<1%	<1%	10±1%	11±1%*
**Total polyunsaturated**	18±1%	15±1%*	18±1%	15±1%*	46±1%	48±1%*

Values are means±SE. * Significantly different from BEFORE, P≤0.05.

Measurements of the following fatty acids represented <1% of the total fatty acid pool or were not detectible in plasma, muscle triglycerides, and muscle phospholipid fractions: C12:0, C20:0, C22:0, C14:1, C20:1, C22:1, C18:3, C20:2, C20:3, C20:5, and C22:6
